# Cardiomyopathy Associated With Tertiary Adrenal Insufficiency Manifesting as Refractory Heart Failure, Shock, and Sudden Cardiac Death: A Case Report

**DOI:** 10.3389/fcvm.2021.720154

**Published:** 2021-11-01

**Authors:** Xuefeng Wang, Yong Luo, Jian Feng

**Affiliations:** Department of Cardiology, The Affiliated Hospital of Southwest Medical University, Luzhou, China

**Keywords:** cardiomyopathy, adrenal insufficiency, corticosteroids, heart failure, sudden cardiac death

## Abstract

Dilated cardiomyopathy is an etiologically heterogeneous disorder. Early diagnosis and prompt treatment of the underlying disease are of great significance. Primary and secondary adrenal insufficiency are considered quite rare causes of dilated cardiomyopathy. However, to the best of our knowledge, no case of cardiomyopathy associated with tertiary adrenal insufficiency has been reported. Herein, we described a 68-year-old woman with a 15-year history of seasonal dermatitis presented with frequent heart failure and shock. At first, she was diagnosed with idiopathic dilated cardiomyopathy, but standard heart failure and antishock treatment failed. Given her long-term use of dexamethasone for treating seasonal dermatitis, and clinical manifestations consistent with adrenal insufficiency, we tested her basal plasma cortisol, simultaneous corticotropin, and other pituitary hormones, confirming that she had tertiary adrenal insufficiency. Additionally, abdominal enhanced computed tomography revealed atrophic bilateral adrenal glands, indicating long-standing and severe adrenal insufficiency. Then hydrocortisone replacement therapy was initiated, and she recovered rapidly. During the next 2 years of follow-up, she never experienced any episodes of heart failure and shock. Unfortunately, she refused the implantation of defibrillator with cardiac resynchronization therapy (CRT-D) and died of sudden cardiac death 2 years later. Although we could not exclude the coincidence of idiopathic dilated cardiomyopathy with tertiary adrenal insufficiency with 100% certainty, her unique clinical course strongly indicated that her cardiomyopathy resulted from tertiary adrenal insufficiency. This case demonstrates that patients on corticosteroids are at risk for tertiary adrenal insufficiency, which may result in refractory cardiomyopathy and even sudden cardiac death.

## Introduction

Adrenal insufficiency is a potentially life-threatening disorder and can be classified as primary, secondary, or tertiary based on its underlying causes (1). Tertiary adrenal insufficiency is the most common form and predominantly results from long-term use of corticosteroids. It is reported that more than 30% of patients receiving corticosteroids may develop tertiary adrenal insufficiency (2). Primary and secondary adrenal insufficiency are considered quite rare causes of dilated cardiomyopathy (3, 4). However, to the best of our knowledge, no case of cardiomyopathy associated with tertiary adrenal insufficiency has been reported in the literature. Herein, we reported an impressive case of dilated cardiomyopathy caused by tertiary adrenal insufficiency manifesting as refractory heart failure, shock, and sudden cardiac death.

## Case Presentation

**Figure 1:**
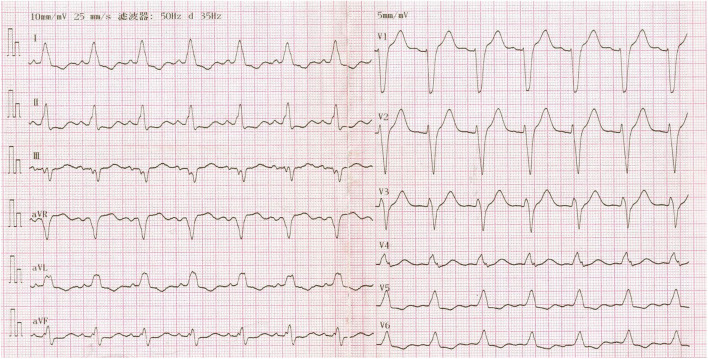
Twelve-lead electrocardiogram showing complete left bundle branch block.

**Figure 2:**
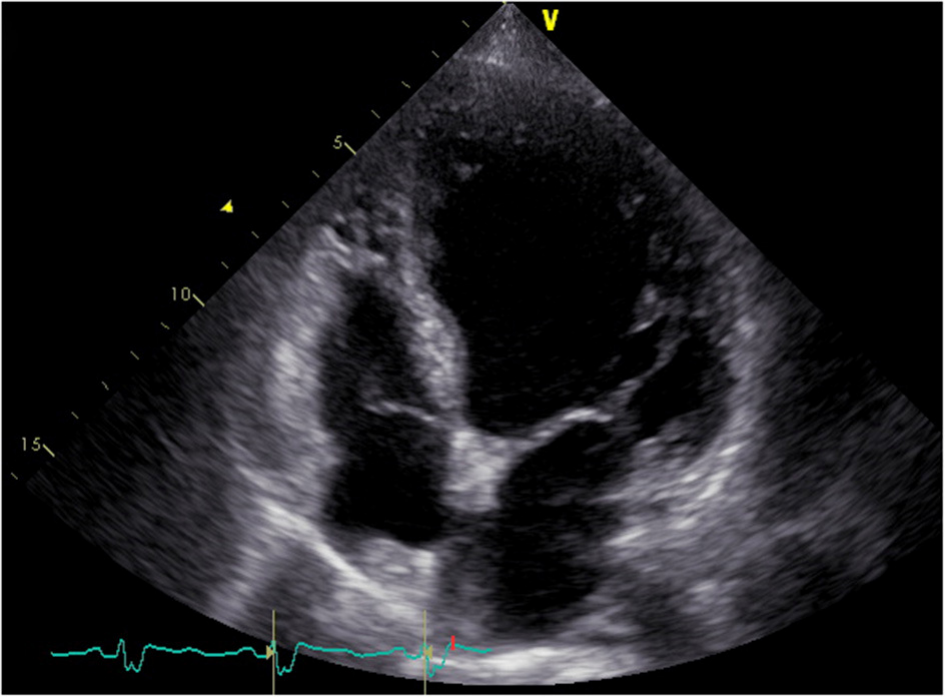
Four-chamber echocardiogram showing a dilated left ventricle.

In April 2018, a 68-year-old woman was referred to our center presenting with rapidly progressive dyspnea. During the previous 12 years, she had been hospitalized more than 100 times for severe heart failure and shock. These episodes were so dramatic that even transient anger could cause acute-onset of dyspnea and shock. Additionally, she reported fatigue, dizziness, anorexia, vomiting, and a 5-kg weight loss during 1 month. Her past medical history revealed a 15-year history of seasonal dermatitis, intermittently treated with oral dexamethasone. On physical examination, she was notable for pallor, hypotension (86/46 mmHg), pulmonary rales, and lower extremities swelling. Laboratory tests were unremarkable except for hyponatremia (Na: 128 mmol/L) and elevated N-terminal pro-B-type natriuretic peptide (NT-Pro BNP: 6,343 pg/mL). In addition, her free thyroxine (FT4) and triiodothyronine (FT3) levels were normal, while thyroid-stimulating hormone (TSH) level was increased (TSH: 11.04 mIU/L, normal TSH: 0.38–5.57 mIU/L). Her electrocardiogram revealed sinus rhythm with complete left bundle branch block (Figure 1). Transthoracic echocardiogram showed a dilated and severely hypokinetic left ventricle (LV 68 mm) with an ejection fraction (EF) of 33% (Figure 2), while coronary angiography revealed no significant coronary artery disease. Therefore, she was diagnosed with idiopathic dilated cardiomyopathy (DCM). However, 3 days after receiving standard heart failure and antishock treatment all her symptoms and signs remained.

**Figure 3:**
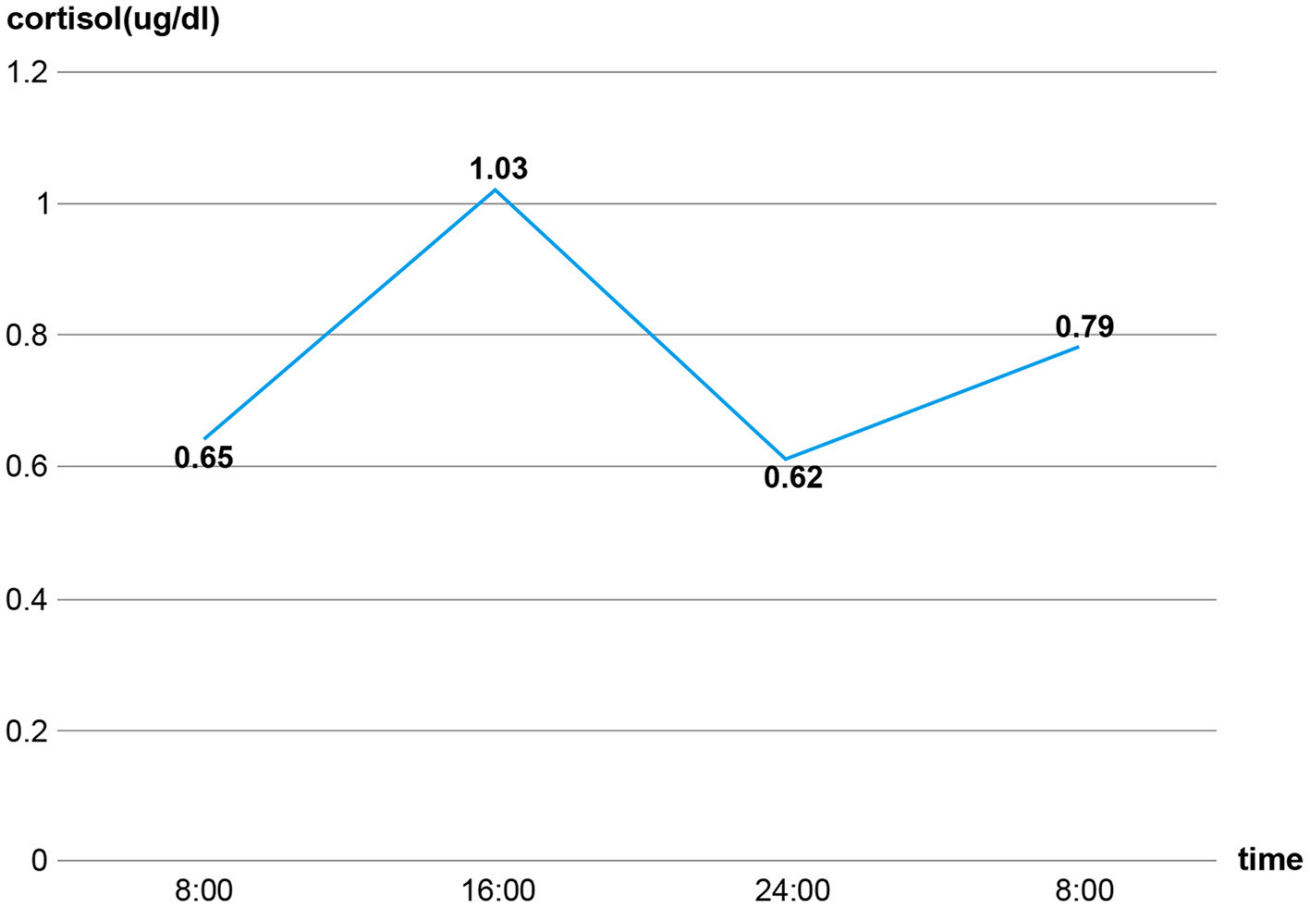
The constantly deficient secretion of cortisol.

**Figure 4:**
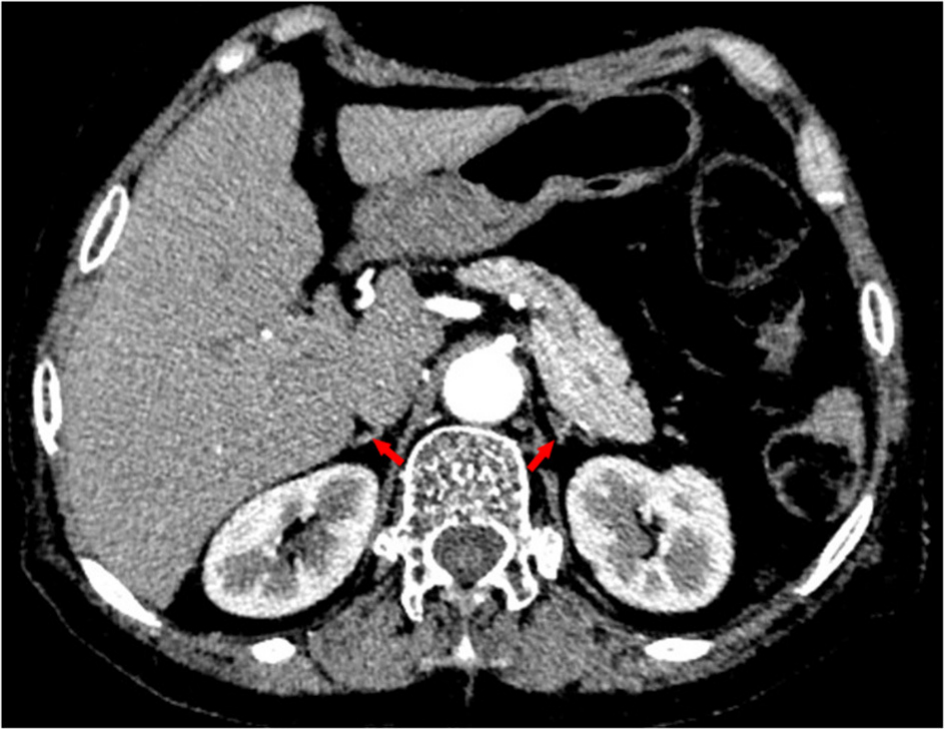
Contrast-enhanced computed tomography revealing atrophic bilateral adrenal glands (red arrows).

Given her long-term dexamethasone treatment and dramatic manifestations consistent with adrenal insufficiency, we tested her basal plasma cortisol and simultaneous corticotropin (ACTH) levels. It turned out that her basal plasma cortisol level was extremely low (0.65 μg/dL; normal 8 a.m. cortisol level: 10.4–26.4 μg/dL) and that ACTH level was low (4.32 pg/mL; normal 8 a.m. ACTH level: 6–40 pg/mL). Continuous cortisol monitoring revealed that her plasma cortisol was constantly deficient (Figure 3). Additionally, her abdominal enhanced computed tomography revealed atrophic bilateral adrenal glands (Figure 4), indicating long-standing and severe adrenal insufficiency. Further autoantibody assays were negative. In addition, her brain magnetic resonance imaging (MRI) and other pituitary hormones (growth hormone, luteinizing hormone, follicle-stimulating hormone, and prolactin) levels were normal. Therefore, the final diagnosis was dilated cardiomyopathy with tertiary adrenal insufficiency.

In addition to previous normal saline, torasemide (10 mg i.v. twice daily), spironolactone (20 mg once daily), digoxin (0.125 mg once daily), dopamine (10 μg/kg/min), and norepinephrine infusion (0.3 μg/kg/min), hydrocortisone (50 mg) was administered intravenously every 6 h. Within 4 days, her blood pressure normalized, physical activity improved, edema resolved completely, and hyponatremia was corrected. Following that, normal saline and norepinephrine were discontinued, dopamine and intravenous hydrocortisone were gradually withdrawn in the next few days, and an oral hydrocortisone maintenance dose was instituted. Two weeks later, she recovered and was discharged on digoxin (0.125 mg once daily), bisoprolol (1.25 mg once daily), spironolactone (20 mg once daily), torasemide (10 mg once daily), and hydrocortisone (12.5 mg + 7.5 mg + 5.0 mg). During the follow-up period, her compliance and persistence with these medications remained excellent, and she had never experienced any episodes of heart failure and shock. We had tried to initiate low-dose candesartan (1 mg once daily) several times but failed, because it caused hypotension. Echocardiogram performed 18 months following hydrocortisone replacement therapy revealed mild improvement in EF and LV reverse remodeling (EF 35%, LV 64 mm), while her complete left bundle branch block remained. Unfortunately, she refused the implantation of defibrillator with cardiac resynchronization therapy (CRT-D) and died of sudden cardiac death in May 2020.

## Discussion

Adrenal insufficiency is a potentially life-threatening disorder characterized by deficient production or action of glucocorticoids due to impairment of the hypothalamic-pituitary-adrenal axis (1). It can be classified as primary, secondary, or tertiary, which result from adrenal gland, pituitary gland, or hypothalamus disorders, respectively (5). Among them, tertiary adrenal insufficiency is the most common form and predominantly results from long-term use of corticosteroids (6). Although higher corticosteroid dose and longer treatment duration convey higher risk, tertiary adrenal insufficiency frequently occurs in patients receiving corticosteroid treatment regardless of administration form, dosing, treatment duration, or underlying disease. It is reported that about 30% of patients receiving corticosteroids may develop adrenal insufficiency (2).

Long-term administration of exogenous glucocorticoids can cause prolonged suppression of hypothalamic secretion of corticotropin-releasing hormone, resulting in deficient production of ACTH and glucocorticoids. Like other forms of adrenal insufficiency, glucocorticoid-induced adrenal insufficiency is associated with a series of non-specific symptoms such as weakness, fatigue, anorexia, abdominal pain, weight loss, and orthostatic hypotension (7). In addition, hyponatremia and elevated TSH are common. However, many patients with this type of adrenal insufficiency may paradoxically exhibit Cushingoid features, and hyperpigmentation is often absent because ACTH is not increased (1). The history of steroid use and clinical characteristics listed above necessitate screening for tertiary adrenal insufficiency. An extremely low basal cortisol level (<3 μg/dL or 83 nmol/L) is sufficient to diagnose adrenal insufficiency. However, basal cortisol concentrations falling in an indeterminate range (3–13 μg/dL or 83–365 nmol/L) should prompt dynamic tests, ideally the standard-dose corticotropin test (8). Dexamethasone is a long-acting steroid and conveys a higher risk for tertiary adrenal insufficiency. For our patient, her long-term use of dexamethasone, peculiar manifestations, along with hormonal tests, confirmed that she had tertiary adrenal insufficiency. Additionally, her adrenal atrophy indicated that she underwent long-standing and severe adrenal insufficiency. Lastly, secondary adrenal insufficiency could be ruled out based on her brain MRI and other pituitary hormones tests results, as secondary adrenal insufficiency results from pituitary disorders and is commonly linked to other pituitary hormone deficiencies.

Notably, cardiovascular manifestations of adrenal insufficiency, including dilated cardiomyopathy, arrhythmias, and Takotsubo cardiomyopathy, were rarely reported in primary and secondary adrenal insufficiency (4, 9, 10). The underlying mechanisms may involve downregulation of adrenergic receptors, membrane calcium transporter dysfunction, decreased phosphorylase activity, increased catecholamine levels, and disturbances in electrolyte levels (11, 12). Furthermore, some patients' cardiac abnormalities may completely resolve after hydrocortisone replacement therapy. It seems that a hypocortisol state may result in cardiomyopathy and/or arrhythmias. Nonetheless, to the best of our knowledge, no case of cardiomyopathy associated with tertiary adrenal insufficiency has been reported in the literature.

DCM is an etiologically heterogeneous disorder. Early diagnosis and prompt treatment of the underlying disease are of great significance (13). For our patient, her long-term use of dexamethasone and adrenal atrophy on computed tomography revealed that she suffered from chronic and severe adrenal insufficiency. More importantly, her first episode of heart failure and shock occurred 3 years after intermittent use of dexamethasone, and these episodes were quite peculiar because even transient anger could cause acute-onset of dyspnea and shock. In addition, standard heart failure and antishock treatment failed, but hydrocortisone replacement therapy dramatically improved her symptoms and echocardiographic parameters. Although we could not exclude the coincidence of idiopathic dilated cardiomyopathy with tertiary adrenal insufficiency with 100% certainty, in the absence of other risk factors for DCM, it is quite reasonable to conclude that our patient's refractory cardiomyopathy resulted from tertiary adrenal insufficiency. For our patient, the cardiac dysfunction caused by tertiary adrenal insufficiency along with superimposed stress precipitated her dramatic and recurrent episodes of heart failure and shock. However, her non-specific symptoms often obscured the underlying etiology, resulting in ongoing adverse left ventricular remodeling. As a result, the damage to her heart was so severe that hydrocortisone and heart failure treatments failed to reverse this process, and she was at growing risk for malignant ventricular dysrhythmia. In this case, CRT-D might be life-saving and improve her prognosis.

In summary, DCM is an etiologically heterogeneous disorder. Early diagnosis and prompt treatment of the underlying disease are of great significance. This case demonstrates that patients on corticosteroids are at risk for tertiary adrenal insufficiency, which may result in refractory cardiomyopathy and even sudden cardiac death.

## Data Availability Statement

The original contributions presented in the study are included in the article/supplementary material, further inquiries can be directed to the corresponding author/s.

## Ethics Statement

Written informed consent was obtained from the patient's daughter for the publication of this case report.

## Author Contributions

XW and YL looked after the patient and wrote the report. JF design the research and took the pictures. All authors have read and approved the final version of the manuscript.

## Funding

This study was supported by Collaborative Innovation Center for Prevention and Treatment of Cardiovascular Disease of Sichuan Province, Southwest Medical University (xtcx2019-13).

## Conflict of Interest

The authors declare that the research was conducted in the absence of any commercial or financial relationships that could be construed as a potential conflict of interest.

## Publisher's Note

All claims expressed in this article are solely those of the authors and do not necessarily represent those of their affiliated organizations, or those of the publisher, the editors and the reviewers. Any product that may be evaluated in this article, or claim that may be made by its manufacturer, is not guaranteed or endorsed by the publisher.

## References

[1] Husebye ES Pearce SH Krone NP Kämpe O. Adrenal insufficiency. Lancet. (2021) 397:613–29. 10.1016/S0140-6736(21)00136-7 33484633

[2] Broersen LH Pereira AM Jorgensen JO Dekkers OM. Adrenal insufficiency in corticosteroids use: systematic review and meta-analysis. J Clin Endocrinol Metab. (2015) 100:2171–80. 10.1210/jc.2015-1218 25844620

[3] Alkhateeb M Alsakkal M Alfauri MN Alasmar D. Reversible dilated cardiomyopathy as a complication of adrenal cortex insufficiency: a case report. J Med Case Rep. (2018) 12:345. 10.1186/s13256-018-1899-1 30458836 PMC6247618

[4] Ikegami Y Fukuda T Jo R Momiyama Y. Reversible cardiomyopathy accompanied by secondary adrenal insufficiency. Circ Heart Fail. (2016) 9:e002919. 10.1161/CIRCHEARTFAILURE.115.002919 26920218

[5] Charmandari E Nicolaides NC Chrousos GP. Adrenal insufficiency. Lancet. (2014) 383:2152–67. 10.1016/S0140-6736(13)61684-0 24503135

[6] Hahner S Ross RJ Arlt W Bancos I Burger-Stritt S Torpy DJ. Adrenal insufficiency. Nat Rev Dis Primers. (2021) 7:19. 10.1038/s41572-021-00252-7 33707469

[7] Chanson P Guignat L Goichot B Chabre O Boustani DS Reynaud R. Group 2: adrenal insufficiency: screening methods and confirmation of diagnosis. Annales d'Endocrinologie. (2017) 78:495–511. 10.1016/j.ando.2017.10.005 29174200

[8] Patti G Guzzeti C Di Iorgi N Maria Allegri AE Napoli F Loche S. Central adrenal insufficiency in children and adolescents. Best Prac Res Clin Endocrinol Metab. (2018) 32:425–44. 10.1016/j.beem.2018.03.012 30086867

[9] Ödek Ç Kendirli T Kocaay P Azapagasi E Uçar T Siklar Z. Acute reversible cardiomyopathy and heart failure in a child with acute adrenal crisis. Paediatr Int Child Health. (2016) 37:148–51. 10.1080/20469047.2015.1120410 27077627

[10] Gotyo N Kida M Horiuchi T Hirata Y. Torsade de pointes associated with recurrent ampulla cardiomyopathy in a patient with idiopathic ACTH deficiency. Endocr J. (2009) 56:807–15. 10.1507/endocrj.K09E-080 19506326

[11] Mozolevska V Schwartz A Cheung D Shaikh B Bhagirath KM Jassal DS. Addison's disease and dilated cardiomyopathy: a case report and review of the literature. Case Rep Cardiol. (2016) 2016:4362514. 10.1155/2016/4362514 28003914 PMC5149592

[12] Campean R Hasun M Stollberger C Bucher J Finsterer J Schnack C. Takotsubo-like syndrome triggered by fludrocortisone overdose for Addison's disease: a case report. J Med Case Rep. (2016) 10:281. 10.1186/s13256-016-1074-5 27729057 PMC5059987

[13] Pinto YM Elliott PM Arbustini E Adler Y Anastasakis A Bohm M. Proposal for a revised definition of dilated cardiomyopathy, hypokinetic non-dilated cardiomyopathy, and its implications for clinical practice: a position statement of the ESC working group on myocardial and pericardial diseases. Eur Heart J. (2016) 37:1850–8. 10.1093/eurheartj/ehv727 26792875

